# Neural Signatures of Cannabis Use: Reversing Cognitive Aging via Whole-Brain Functional Network Connectivity

**DOI:** 10.21203/rs.3.rs-6977015/v1

**Published:** 2025-08-01

**Authors:** Zening Fu, Kent Hutchison, Armin Iraji, Jing Sui, Vince Calhoun

**Affiliations:** Georgia Institute of Technology, Emory University and Georgia State University; University of Colorado Anschutz Medical Campus; Tri-Institutional Center for Translational Research in Neuroimaging and Data Science (TReNDS); University of Chinese Academy of Sciences; Georgia Institute of Technology

## Abstract

Given the growing trend toward permissive societal attitudes and the legalization of cannabis, coupled with an increasing recognition of its therapeutic potential, there has been a notable rise in cannabis consumption among older adults. Cognitive aging, one of the most prevalent concerns in this demographic, intersects with cannabis use, which shares several neural correlates. However, the precise impact of cannabis on the aging brain and cognitive function remains poorly understood. In this study, we leveraged large-scale data from the UK Biobank, which includes over 25,000 participants, to conduct a comprehensive examination of the relationships between cannabis use, normative aging, and cognitive function. Our focus was on how these factors correlate with brain functional network connectivity (FNC), aiming to elucidate the interactive effects underlying brain neuroimaging patterns. Our findings reveal that cannabis usage and healthy aging are associated with overlapping brain network configurations, particularly within the FNC between subcortical and sensorimotor regions, as well as between subcortical and cerebellar areas, albeit with significantly reversed effects. Notably, cannabis users exhibited superior performance across multiple cognitive domains, and interestingly, the effects of cannabis and cognition are presented concurrently across a range of brain systems. In conclusion, our study offers valuable insights into the potential influence of cannabis on brain aging and cognitive performance. The results suggest that cannabis users display brain network characteristics typically associated with younger brains, along with enhanced cognitive abilities, highlighting a potential modulatory role for cannabinoids and endocannabinoids in neurodegenerative processes, as explained through neural dedifferentiation and compensation theories.

## Introduction

Cannabis remains the most widely used illicit substance globally. It has traditionally been associated with a range of adverse outcomes, including elevated risks of academic underachievement, unemployment, psychiatric disorders, suicidal ideation, cannabinoid hyperemesis syndrome, and cardiovascular complications^1^. However, in recent years, shifting societal norms, increasing legalization, and growing recognition of cannabis’s therapeutic potential have led to a marked rise in its consumption^2–4^. Currently, cannabis ranks as the third most commonly used psychoactive substance, following alcohol and tobacco^5^. Importantly, older adults represent the fastest-growing demographic of cannabis users^6^. This population is increasingly turning to cannabis as a potential intervention for managing chronic physical and mental health conditions^7^. Over the past decade, self-reported cannabis use among older individuals has risen substantially, with prevalence rates ranging from 10.0–11.9% among those aged 45–64 and from 4.1–5.9% among those aged 65 and above^8^. Given the distinct neurobiological, physiological, and psychosocial changes that accompany aging, the effects of cannabis in older adults may differ significantly from those observed in younger populations. These age-related changes may modulate both the pharmacodynamics and the functional outcomes of cannabis use. Therefore, there is a pressing need for rigorous, systematic research to evaluate the impact of cannabis consumption in later life, particularly concerning its effects on key domains of normative aging.

Cannabis use leads to significant alterations in both brain structure and function across diverse populations. These neural changes encompass modifications in gray matter and white matter volumes, cortical thickness, and patterns of functional activation and connectivity observed during both taskbased and resting-state neuroimaging^9–13^. For example, even minimal exposure—such as one or two instances of cannabis use—has been linked to increased gray matter volume in regions including the bilateral medial temporal lobes, posterior cingulate cortex, lingual gyri, and cerebellum among adolescents^14^. Notably, some of these changes have been linked with variations in cognitive reasoning and the emergence of anxiety-related symptoms later in life. A recent systematic review synthesizing findings from 21 neuroimaging studies on cannabis use reported that the majority of investigations identified increased resting-state functional connectivity (FC) among cannabis users, particularly within frontal brain networks^12^. These networks are critically involved in a wide range of cognitive functions, suggesting that cannabis use may modulate large-scale brain systems related to executive processes, attention, and affect regulation.

Cognitive aging represents a critical dimension of brain health, with substantial implications for the daily functioning and quality of life in older adults. Emerging findings from cognitive neuroscience have demonstrated that both normative aging and individual differences in cognitive performance are associated with reorganization within overlapping large-scale brain networks^15–17^. These patterns suggest that neuroimaging can serve as a reliable modality for characterizing the neural substrates of cognitive aging and tracking changes in brain function throughout the aging process. Given the known influence of cannabis on neural systems, it is plausible that cannabis use may interact with mechanisms underlying brain aging and cognitive performance. Neuroimaging offers a powerful approach for disentangling these complex relationships. Prior research has shown that cannabis use is associated with alterations in brain activity across multiple cognitive domains, including memory processing^18^, social and emotional regulation^19^, and executive functioning^20,21^. A deeper understanding of the neurobiological effects of cannabis on cognitive functioning across the aging spectrum is essential for informing the development of targeted, evidence-based interventions. Such insights may ultimately contribute to the use of cannabinoid-based therapies aimed at mitigating or potentially reversing age-related cognitive decline.

Several studies have explored the effects of cannabis on brain function and cognitive aging, but the findings are often inconclusive and sometimes contradictory^6,22,23^. For instance, several investigations have reported that cannabis users show diminished FC within the frontal network and between the frontal and temporal networks^13,24,25^, while others have observed increased FC among similar areas associated with cannabis usage^26,27^. A recent review highlighted significant heterogeneity and methodological limitations in the existing body of research on cannabis use and cognitive function, suggesting that definitive conclusions regarding its effects on the brain remain elusive^28^. One plausible explanation for these inconsistencies is the frequent use of small sample sizes in earlier studies, which may lack sufficient statistical power to accurately capture the real associations between cannabis consumption and brain function. Indeed, the effect sizes between behavioral phenotypes and brain activity are often smaller than anticipated, with false negative rates remaining high (50%−75%) even in studies with samples as large as 2,000 participants^29^.

Furthermore, while existing studies have concentrated on structural brain markers, functional neuroimaging techniques have been relatively underutilized in exploring the complex relationships between cannabis use, brain aging, and cognitive decline. Neuroimaging research has demonstrated that functional imaging is more sensitive to changes than anatomical imaging^30^, with stronger associations to cognition and greater disruption in brain disorders^31^. However, the connections between brain age, cognition, and cannabis use have yet to be comprehensively examined, particularly in terms of their shared and distinct neural representations as captured by functional neuroimaging methods. Therefore, large-scale, thorough investigations such as the present study, which encompasses over 25,000 participants, can provide invaluable insights into the effects of cannabis on cognitive aging. This research has the potential to enhance our understanding of the neural mechanisms that link functional brain organization to cannabis use in the context of normal aging.

Leveraging large-scale neuroimaging and behavioral data from the UK Biobank (UKB) cohort, the present study employed a fully automated, data-driven analytical framework, namely NeuroMark, to investigate connectome-based signatures associated with cannabis use, brain aging, and cognitive performance. The primary objective was to elucidate the impact of cannabis use on healthy cognitive aging in older adults. By extracting and comparing correlated FC patterns and their associated effect maps, we aimed to quantify the extent to which cannabis use and cognitive aging converge within the brain’s functional architecture. We hypothesized that cannabis use may influence brain aging and cognitive function by modulating the organization of large-scale functional networks. Specifically, we posited that cannabinoids and endocannabinoids exert neuroprotective effects, potentially supporting cognitive resilience and mitigating age-related neurodegeneration. These effects may operate through mechanisms such as neural dedifferentiation and compensatory reorganization of brain networks, reflecting an adaptive process that sustains cognitive performance in the aging brain.

## Materials and Methods

### Participants

UKB is a large-scale, ongoing, longitudinal cohort study designed to investigate a wide range of genetic, environmental, and lifestyle factors influencing health outcomes. Initiated in 2006, the study has recruited over 500,000 participants from across the United Kingdom, collecting extensive phenotypic, clinical, and behavioral data. In 2014, a subset of participants began undergoing multimodal brain imaging, referred to as the “imaging visit,” with follow-up imaging (the “first repeat imaging visit”) commencing in 2019 for a portion of this cohort. The present study, conducted under UKB Application ID 34175, utilized imaging and behavioral data from a subset of 37,929 participants aged between 44 and 81 years. This curated dataset enabled robust analysis of functional brain networks in relation to cannabis use, brain aging, and cognitive function.

### Preprocessing

We obtained preprocessed resting-state functional MRI (rsfMRI) data from the UKB Accelerating Medicines Partnership (AMS) server (https://ams.ukbiobank.ac.uk/ams/). We performed spatial normalization to align the rsfMRI volumes with the standard echo-planar imaging (EPI) template space^32,33^. Spatial smoothing was then applied using a Gaussian kernel with a full width at half maximum (FWHM) of 6 mm to enhance the signal-to-noise ratio. To ensure the integrity and reliability of the imaging data, we implemented a rigorous quality control (QC) pipeline based on the NeuroMark framework^33^. This procedure involved a thorough assessment of both spatial and temporal properties of the rsfMRI data. Scans exhibiting excessive head motion—defined as rotational displacements greater than 3 degrees, translational movements exceeding 3 mm in any direction, or a mean framewise displacement (FD) exceeding 0.35 mm—were excluded from further analysis. Additionally, to verify spatial quality, we assessed the similarity between each scan’s brain mask and the group mask, excluding scans that fell below predefined correlation thresholds.

For participants with two imaging sessions that passed QC, only the first scan was retained. This QC protocol, which has been validated and employed in our previous work^34–38^, ensures the robustness and fidelity of the imaging data by minimizing confounding influences from motion artifacts and preprocessing anomalies. More details regarding the preprocessing and quality control procedures are provided in the supplementary material sections “Minimal Preprocessed Data and Normalization” and “Quality Control for the rsfMRI Data”.

### Phenotypic Information and Cognitive Assessments

The primary variable of interest in this study was lifetime cannabis use, derived from UKB Data-Field 20453 (“ever taken cannabis”). Participant answers to the question, “Have you used cannabis (marijuana, grass, hash, ganja, blow, draw, skunk, weed, spliff, dope), even if it was a long time ago?” were categorized as follows for the present study: none-users (0 time) and cannabis users (at least 1 time). The cannabis users were also divided into subgroups: light users (1 ~ 10 times) and heavy users (more than 10 times). Additional covariates included participant age at the time of the imaging session (Data-Field 21003) and biological sex (Data-Field 31). Sex information was obtained from the central NHS registry at the time of recruitment and may include a combination of registry-based and self-reported data. These variables were incorporated to control for demographic variation and to explore potential moderating effects in the relationship between cannabis use, brain function, and cognitive aging.

For the cognitive assessments, we utilized the UKB cognitive battery^39^, which encompasses a series of tasks designed to evaluate cognitive functioning across multiple domains, including executive function, memory, reasoning, and vocabulary. These domains are particularly pertinent given their potential association with cannabis use and its neurocognitive implications. We selected nine cognitive tasks that provide comprehensive coverage and align with the objectives of the present study: (1) reaction time test, (2) fluid intelligence test, (3) numeric memory test, (4) trail-making test, (5) symbol digit substitution test, (6) picture vocabulary test, (7) paired associate learning test, (8) tower rearranging test, and (9) matrix pattern completion test. Detailed descriptions and related data fields of each task are provided in the supplementary material section “UK Biobank Cognitive Assessments”.

### NeuroMark to Extract Functional Network Connectivity

#### NeuroMark to Extract Functional Network Connectivity

We employed the fully automated Independent Component Analysis (ICA) framework “NeuroMark” to extract individual-level FC features. Specifically, we utilized the NeuroMark 1.0 functional template as a spatial prior in a spatially constrained ICA, enabling the estimation of subject-specific intrinsic connectivity networks (ICNs) and their associated time courses (TCs). Following decomposition, we conducted post-processing of the TCs and calculated functional network connectivity (FNC) using Pearson correlation coefficients between the post-processed TCs of all network pairs, yielding subjectlevel whole-brain FNC matrices. These FNC values were subsequently standardized via z-scoring, facilitating cross-subject comparability. NeuroMark provides a robust hybrid framework that ensures consistency of extracted features across individuals and imaging sessions while also accounting for inter-individual variability in single-scan estimates. Its effectiveness in identifying reproducible and biologically meaningful neuroimaging biomarkers has been validated across a wide range of studies and clinical populations^35,40–44^. Additional details about the NeuroMark framework and FNC estimation can be found in the supplementary material section “NeuroMark Framework and Functional Network Connectivity.”

### Associations Between FNC and Cannabis Use, Age, and Cognition

To examine FNC alterations associated with cannabis use, we employed a linear mixed-effects model (LMM) for each pairwise FNC measure. This modeling approach is well-established in large-scale neuroimaging analyses and has demonstrated robust performance in identifying brain-behavior relationships across diverse populations^45–47^.

In our primary analysis, each FNC pair served as the dependent variable, with cannabis use included as a fixed effect. Additional covariates—age, sex, and mean FD—were also incorporated as fixed effects to control for demographic and motion-related confounds. To further investigate normative aging effects on FNC, we conducted a separate LMM analysis restricted to non-users, modeling each FNC pair as a function of chronological age, with sex and mean FD included as covariates. In addition, to evaluate whether cannabis use is associated with differences in cognitive performance, we first conducted twosample t-tests comparing cognitive assessment scores between cannabis users and non-users while controlling for age, sex, and mean FD as covariates. Subsequently, to investigate the neural correlates of cognitive performance independent of cannabis exposure, we applied the LMM within the non-user subgroup. Each pairwise FNC measure was modeled as the dependent variable, and each cognitive assessment score was a fixed effect. Age, sex, and mean FD were also included as fixed covariates to account for potential demographic and motion-related confounding factors.

For each LMM analysis, we computed the correlation (*r*), t-statistic (*t*), and effect size (Cohen’s *d*) to quantify the strength and direction of the associations between FNC and the variables of interest—namely, cannabis use, chronological age, and cognitive performance. To control for the inflated risk of Type I error due to multiple comparisons across numerous FNC pairs, we applied false discovery rate (FDR) correction using the Benjamini–Hochberg procedure^48^. This approach ensured rigorous statistical inference while maintaining an acceptable false-positive rate across the high-dimensional connectivity data.

### Correlative Network Anatomy and Overlap between Models

The correlative relationship between each FNC and factors such as cannabis use, age, and cognitive performance can be quantified using the t-value estimated from the LMM. For each association model—specifically, the associations between FNC and cannabis use, age, or each cognitive assessment—we generated an FNC-level representation based on the aggregated t-values. To identify functional domains that play a significant role in explaining these correlative relationships, we grouped the ICNs into seven functional domains according to the NeuroMark 1.0 functional template definition. For each pair of functional domains, we summed the t statistics of all FNC pairs and then normalized this sum by the total number of FNCs belonging to that domain pair^49^. This normalization helps control for the influence of domain size. After obtaining these FNC- and domain-level representations, we evaluated the similarity and distinction of the correlative models. We calculated the Pearson correlation between the t-value maps from the cannabis-use model and those from the age model and each cognitive assessment model, at both the FNC and functional domain levels.

### Code Availability

The NeuroMark framework, along with its templates, has been fully integrated into the Group ICA of fMRI Toolbox (GIFT, https://trendscenter.org/software/gift/), which is publicly available for download and use by the global research community. Additional code and analytical resources used in the present study are available upon reasonable request from the corresponding author.

## Results

### Brain Parcellation

NeuroMark QC identified 23970 subjects who had at least one valid scan to investigate FNC associated with cannabis use. Among these subjects, we included 18482 non-users for the association analysis between FNC and age. The completion rates for various cognitive assessments differed, resulting in 11586 to 17146 non-users available for the association analyses between FNC and different assessments. Detailed demographic information is provided in Table 1. Assuming moderate observational errors (approximately 5% of the mean) in neuroimaging data and weak effects (with a correlation coefficient of r = 0.1), a sample size of > 11586 subjects is sufficient to achieve a power of 0.80 and a Type I error rate of 0.05 in the association analysis. The 53 ICNs identified by the NeuroMark 1.0 functional template were categorized into seven functional domains, as illustrated in Table S1 and Figure S1. This classification was based on the AAL atlas and our previous research^50^. The seven domains are as follows: subcortical (SC), auditory (AUD), visual (VS), sensorimotor (SM), cognitive control (CC), default mode (DM), and cerebellar (CB).

### Cannabis Use associated FNC Changes

**Figure 1:**
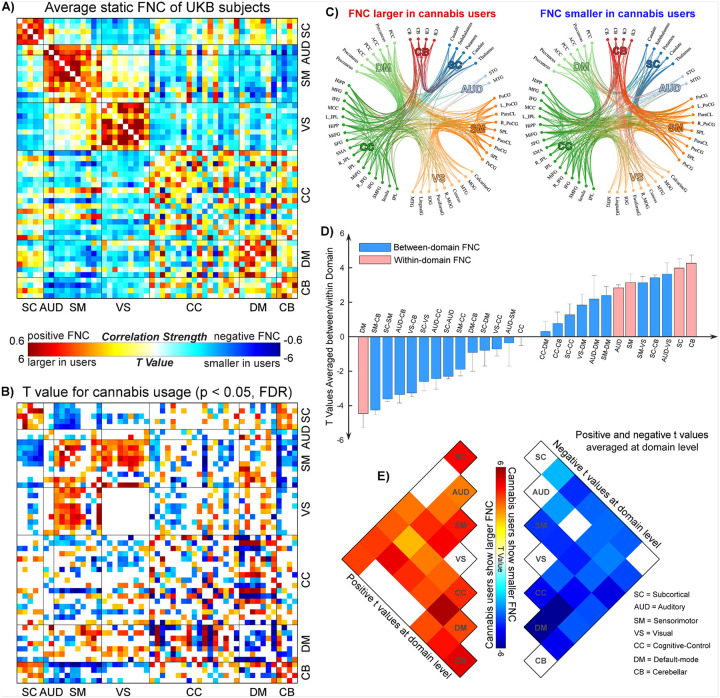
NeuroMark FNC and the FNC changes associated with cannabis use in older adults. **A)** Averaged FNC patterns across UKB subjects extracted by the NeuroMark framework. 53 ICNs are ordered into 7 functional domains for the presentation. **B)** Significant t values from the LMM analysis of the FNC changes associated with cannabis use (p < 0.05, FDR corrected). Warm color indicates larger FNC in cannabis users, while cool color indicates smaller FNC in cannabis users. **C)**Circular plots show the FNCs that are significantly larger and smaller in the cannabis group, respectively. **D)** Distributions of t values averaged at the domain level. For each pair of domains (within-domain and between-domain), we averaged the t values of all FNCs belonging to that domain pair. **E)** The cell plots show the domain-level representation of t values with positive and negative weights, respectively. Positive and negative values were summarized separately for each domain pair to demonstrate their relative contribution.

Figure 1 illustrates the findings on FNC and its relationship with cannabis use in the UKB samples. In Fig. 1A, we show the averaged FNC patterns across cannabis users and non-users. The results reveal modular patterns within each functional domain, consistent with previous research on other datasets. This consistency supports the validity of applying the NeuroMark framework to the UKB data. Figure 1B presents the significant t-statistics from the LMM analysis that compares FNC between cannabis users and non-users (p < 0.05, FDR corrected). Figure 1C employs circular plots to illustrate the increases and decreases in FNC associated with cannabis use. Cannabis use had varied effects on whole-brain FNC. Specifically, cannabis users exhibited increased FNC within the SC, AUD, SM, and CB domains. In contrast, users showed decreased FNC in the DM domain. The impact of cannabis on FNC within the CC domain is mixed, with both increases and decreases in connectivity noted. Additionally, cannabis use did not appear to affect any FNC in the VS domain. When examining the connectivity between different domains, cannabis users showed decreased FNC between the SC/CB and SM/VS domains but increased FNC between the SC and CB domains. The FNC between the CC and DM domains exhibited heterogeneous changes in cannabis users. Figures 1D and 1E summarize the positive and negative t-values at the domain level. Overall, these findings align with the observation at the FNC level that within-domain FNC tends to increase in cannabis users, except in the DM and VS domains.

### Cannabis and Normal Aging: FNC as a Neural Substrate of Association

**Figure 2:**
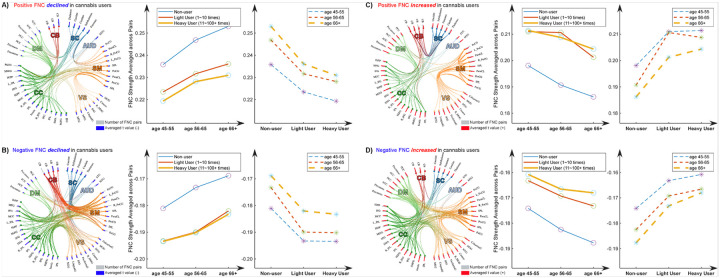
Trajectory of FNC associated with cannabis usage and during normal aging. FNCs with significant alterations in the cannabis users were divided into 4 groups according to their signs and changing directions. Subjects were divided into subgroups according to their cannabis usage (non-users: 0 time; light users: 1–10 times; heavy users: 11+ times) and age (45–55 years; 56–65 years; 66+ years). **A)** The overall FNC was summarized across 125 positive FNCs, which were lower in the cannabis group. **B)** The overall FNC was summarized across 195 negative FNCs, which were lower in the cannabis group. **C)** The overall FNC was summarized across 138 positive FNCs, which were higher in the cannabis group. **D)** The overall FNC was summarized across 183 negative FNCs, which were higher in the cannabis group. Cannabis use appears to have a generally opposite effect on FNCs compared to normal aging.

To further probe the relationship between cannabis use and FNC alterations, we categorized the significant FNC into four groups based on their signs and the direction of change. Specifically, we found that 125 pairs of positive FNC and 195 pairs of negative FNC decreased among cannabis users, and 138 pairs of positive FNC and 183 pairs of negative FNC increased in this group. We then segmented the subjects into different age groups (45–55 years, 56–65 years, and 66 + years). We averaged the t-values within each group across FNC pairs and subjects, and the results are displayed in Fig. 2. Figure 2A illustrates the findings for the positive FNC that declined in cannabis users. Interestingly, we observed opposing effects between cannabis use and aging on FNC. Increased cannabis usage was associated with a decrease, while normal aging correlated with an increase in these pairs of FNC. Similar patterns were noted in the other groups (see Figs. 2B, C, and D), where cannabis use and normal aging exhibited reversed effects on FNC alterations.

**Figure 3:**
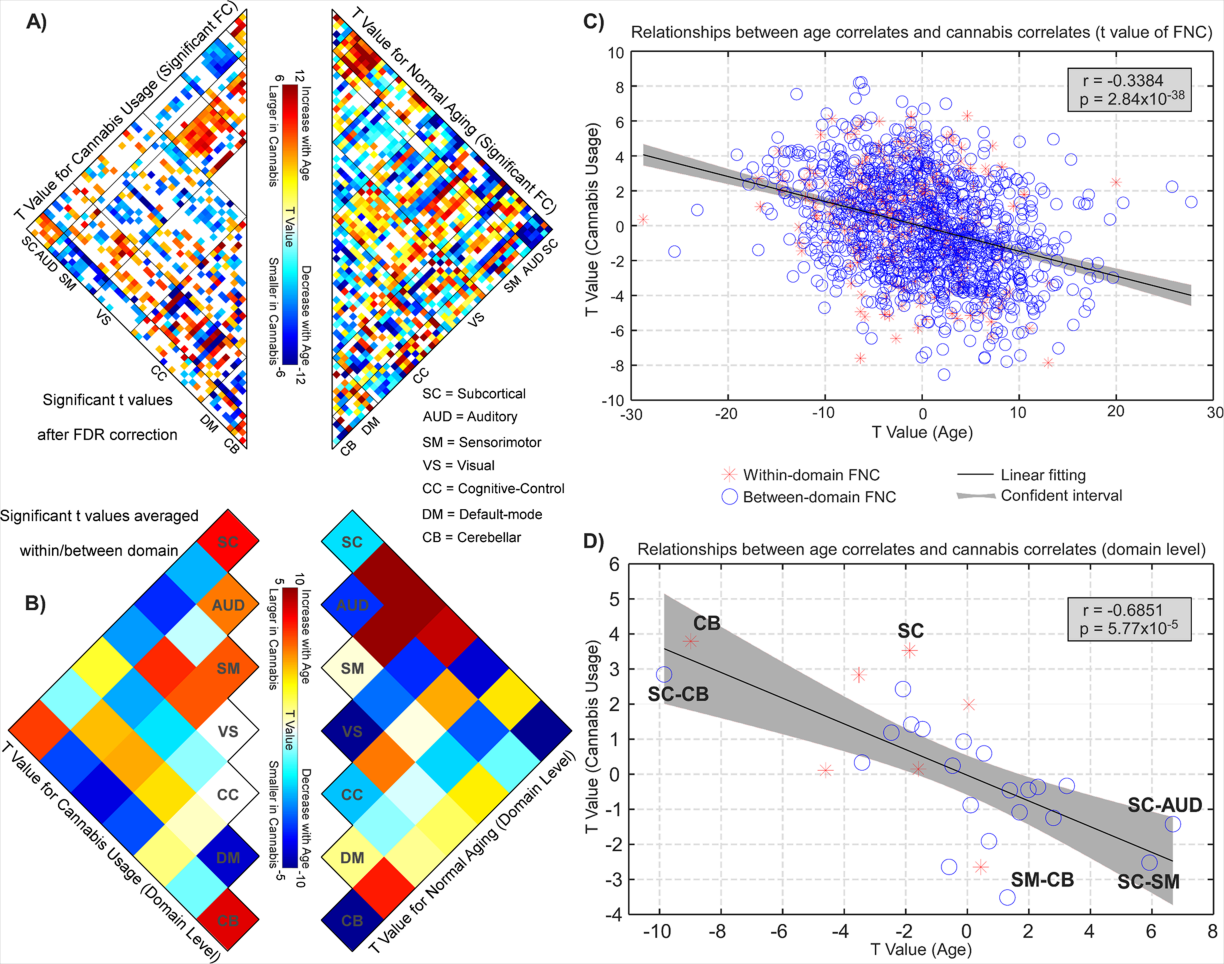
Overlapping FNC changes associated with cannabis use and normal aging. **A)** The left matrix shows the t values with significant FNC changes associated with cannabis use, while the right matrix shows the t values with significant FNC changes associated with normal aging. **B)** The cell plots show the domain-level representation of t values associated with cannabis use and normal aging. **C)** The scatter plot is the representation of the correlation between t values associated with cannabis use and normal aging. A significantly negative correlation is identified between t statistics of cannabis use and the t statistics of normal aging at the FNC level (r = −0.3384, p = 2.84 × 10^−38^, n = 1378). **D)** T values from each model were summarized for domain pair. A significantly negative correlation is identified between t statistics at the domain level (r = −0.6851, p = 5.77 × 10^−5^, n = 28).

Figure 3 presents the results of the associations between age and FNC, offering additional evidence that cannabis use was linked to normal aging in this context. A summary of the t-maps from low-level FNC (Fig. 3A) to high-level domains (Fig. 3B) revealed significant trends. The FNC and functional domains that showed a significant association with cannabis use also demonstrated considerable involvement in the aging process, albeit in contrasting directions. That is, FNC patterns that increased or decreased in strength with aging were lower or higher among cannabis users, respectively. For instance, the FNC between the SC and SM domains was diminished in cannabis users, while it strengthened with aging. Conversely, the FNC between the SC and CB domains was elevated among cannabis users but decreased in strength as age increased. At the FNC level, the t-values associated with cannabis use and age were inversely correlated (r = −0.3384, p < 10^− 5^, Fig. 3C). When summarizing the whole-brain connectome in the functional domain representations, we noted a generally elevated correlation between models related to cannabis use and aging (r = −0.6851, p < 10^− 4^, Fig. 3D). Notably, FNC within the CB domain, as well as the connectivity between SC and CB domains, exhibited the most positive t-values associated with cannabis use (higher in users) and the most negative t-values with aging (declining across age). In contrast, the FNC between SC and AUD/SM domains and between the SM and CB domains displayed the most negative t-values linked to cannabis use (lower in users) and the most positive t-values associated with aging (increasing with age).

### Cannabis and Cognition: FNC as a Neural Substrate of Association

**Figure 4:**
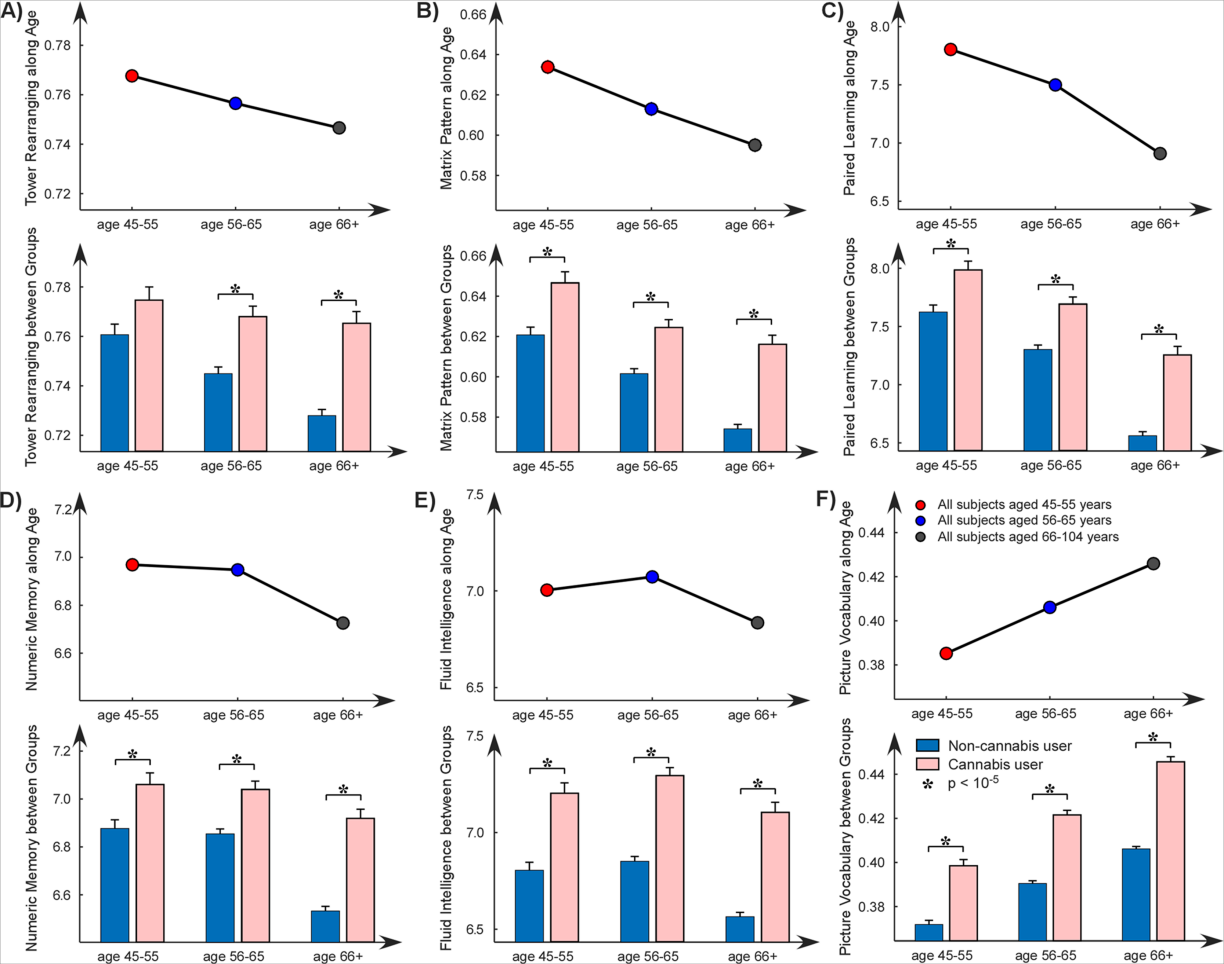
Cannabis use is associated with differences in cognitive functioning during normal aging. Cognitive performance in 6 functional domains, including **A)** Tower rearranging task, **B)** Matrix pattern completion task, **C)** Paired associate learning task, **D)** Numeric memory task, **E)** Fluid intelligence task, and **F)** Picture vocabulary task are examined and displayed. The upper panel of each subplot shows an overall decreasing trend in cognitive performance, except for the picture vocabulary task. The bar plots in the lower panel reveal a positive effect introduced by cannabis use on cognitive function, where cannabis users show generalized better cognitive performance in different age populations.

Normal aging typically involves a gradual decline in cognitive abilities, particularly in processing speed, working memory, and executive function. As illustrated in Fig. 4, six out of nine cognitive assessments showed significant effects with normal aging, where five scores declined with aging and the picture vocabulary score increased with aging. When we divided the subjects into cannabis users and non-users, we still observed similar trends in cognitive decline/increase associated with aging. Furthermore, we found that cannabis use had positive effects on most cognitive functions compared to normal aging. Specifically, cannabis users performed better in various cognitive tasks, including tower rearranging, matrix pattern completion, paired associate learning, numeric memory, fluid intelligence, and picture vocabulary. Additionally, these effects—where cannabis users outperform non-users—were evident across different age groups (45–55 years, 56–65 years, and 66 + years).

**Figure 5:**
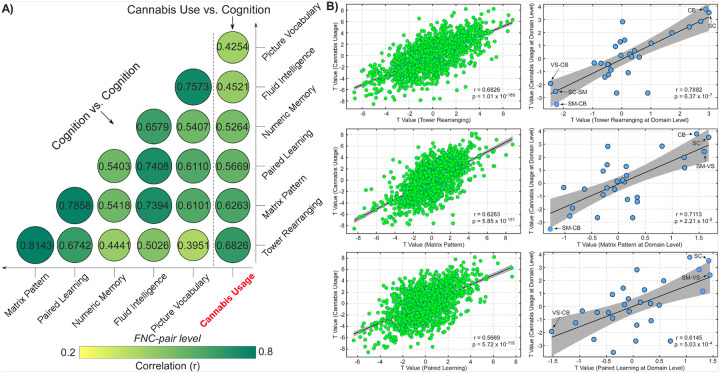
Overlap of correlation models between cannabis use and cognitive function during normal aging. **A)** To evaluate the extent to which their FNC correlates were similar to or distinct from each other, we calculated the Pearson’s correlation between t values from the LMM for cannabis use and the t values for each of the cognitive tasks at the FNC level. All correlations are significant at p < 10^−5^. **B)** The scatter plots show three examples of the positive correlation between cannabis use model and cognition model. The first column is the representation of the correlation at the FNC level (n = 1378), and the second column is the representation of the correlation at the domain level (n = 28).

The results from the LMM on the cognitive assessments offer further evidence of the connection between cannabis use and cognition, particularly in relation to functional brain imaging. After eliminating confounding effects caused by cannabis use, we found that cognitive assessments were significantly associated with FNC across the entire brain (p < 0.05, FDR corrected). To further elucidate the relationship between cannabis use and cognition at the neural level, we examined the overlap in FNC models associated with cannabis use and cognitive performance. We discovered positive correlations between the t-values for cannabis use and cognitive performance at the FNC level (r = 0.4254 to 0.6826, p < 10^− 5^, Fig. 5A). The strongest correlation was between cannabis use and performance on the tower rearranging task (r = 0.6826, p = 1.01 × 10^− 189^). These positive correlations in connectivity t-values between cannabis use and cognitive assessments support their behavioral relationship. Given that the cognitive models were similar (r = 0.3951 to 0.8143), we presented three examples of correlation effects between cannabis use and cognitive function for simplicity in Fig. 5B. Domain-level analysis revealed similar correlation patterns aligned with the FNC-level results (r = 0.6145 to 0.7882, p < 10^− 4^). FNC within the SC and CB domains, as well as between the SM and VS domains, demonstrated the highest positive t-values linking to cannabis use and cognitive performance. In contrast, FNC between the CB and VS/SM domains, as well as between the SC and SM domains, exhibited the highest negative t-values associated with cannabis use and cognitive performance. Taken together, these findings indicate that cannabis use modulates FC in brain networks that are critically involved in cognitive functioning.

## Discussion

In the present study, we investigated the effects of cannabis use on functional brain connectivity and its relationship to cognitive aging, a critical issue in geriatric neuroscience. Our results demonstrated significant associations among cannabis use, chronological age, and cognition, as reflected in overlapping widespread alterations in brain network connectivity. These findings suggest that cannabis use may be associated with a deceleration of neural aging processes and the preservation of cognitive function in older adults. Converging neural patterns linked to cannabis use, aging, and cognition were observed, indicating overlapping connectivity signatures. Specifically, cannabis use was associated with increased FC within and between subcortical and cerebellar regions, enhanced connectivity between sensorimotor and visual networks, and reduced connectivity between subcortical/cerebellar regions and sensory cortices. These connectivity profiles may reflect a potential neuroprotective or compensatory effect of cannabis, potentially mitigating age-related neural dedifferentiation^15,51^. Collectively, these findings provide novel insights into the complex and multifaceted relationships among cannabis use, cognitive aging, and the organization of large-scale brain networks.

### Cannabis, Brain Aging Deceleration and Cognitive Preservation in Elderly

Cannabis has been utilized for millennia across medicinal, spiritual, and recreational contexts. In contemporary discourse, however, it is frequently associated with adverse health outcomes, particularly those involving neural function. Cannabis-related alterations in mood, cognition, and perception can lead to elevated risks for psychiatric conditions, including depression, anxiety, suicidal ideation, and psychosis^52,53^. During recent years, legislative reforms and increasing recognition of the therapeutic potential of cannabis have contributed to a marked rise in its use among older adults. Unlike other populations, the effects of cannabis in this population may be complicated by age-related factors, especially for the changes in brain morphology and function found to be affected by cannabis use in younger populations^6,54^. This study builds on prior work documenting substantial variability in the relationship between cannabis use and the functioning brain^55^. Despite growing interest, empirical investigations examining the impact of cannabis on the aging brain remain scarce and often yield inconsistent findings. To address this gap, in this study, we capitalized on advances in a fully automated framework to achieve an individual-level estimation of whole brain FC in a purely data-driven manner and comprehensively characterize how they are associated with cannabis use via a widely used LMM approach^36,45,56,57^.

To date, the majority of human neuroimaging studies investigating the effects of cannabis on brain function have focused on adolescents and young adults^12,23^. In contrast, research examining cannabis use in older adults remains limited, and existing findings are marked by considerable variability and inconsistency. For instance, a study examining adults aged 19 to 55 years reported that cannabis users exhibited reduced FC between the amygdala and cuneus, as well as between the cingulum and occipital and temporal cortices, with no significant group differences observed in hippocampal connectivity^58^. In another investigation, cannabis users demonstrated increased FC within both the salience network and the default mode network (DMN), alongside anticorrelated FC between these two networks^59^. However, these findings diverge markedly from those of a study specifically targeting older adults (ages 60–88), which reported increased FC between the cerebellum and hippocampus in cannabis users—suggesting a potential neuroprotective effect in aging^23^. Such discrepancies across studies may stem, in part, from limited sample sizes, which constrain statistical power and increase the risk of inflated or unreliable brain-behavior associations. Given the need for larger, well-powered datasets to establish robust neurobiological correlates of cannabis use, the UKB offers a valuable resource for systematically investigating the effects of cannabis on the aging brain in a more reliable and generalizable manner.

The observed cannabis-related enhancement of FNC within cortical-subcortical-cerebellar circuits aligns with anatomical evidence indicating high concentrations of cannabinoid receptors in these regions^60,61^. The endocannabinoid system (ECS), a complex cell-signaling network, plays a critical role in regulating numerous physiological processes^62^ and undergoes age-dependent changes that may modulate the body’s response to cannabinoids such as delta-9-tetrahydrocannabinol (THC) and cannabidiol (CBD)^63,64^. The ECS is intricately involved in aging, with cannabinoid receptors regulating cellular mechanisms underlying age-associated inflammation. Notably, older adults exhibit reductions in both cannabinoid receptor density and endogenous endocannabinoid levels, which may contribute to the cognitive decline commonly observed with aging^65^. Preclinical studies have demonstrated age-specific interactions between cannabinoids and the ECS; for instance, administration of low-dose THC in aged animals would improve spatial memory, enhance synaptic density, and reduce neuroinflammation—potentially through reactivation of hypoactive cannabinoid receptor circuits^66^. THC-induced activation of microglia within the receptors-expressed brain regions, along with subsequent neuroinflammatory responses, has been implicated as a potential mechanism underlying impairments in classical conditioning observed in animal models^67^. It is also believed that the activation of cannabinoid receptors restores FC in networks particularly susceptible to age-related declines, such as the cerebellum-subcortical-cortical pathways^68,69^. Consequently, our findings of increased FC in brain regions with high cannabinoid receptor expression may reflect the modulatory influence of cannabinoids on ECS activity, potentially facilitating neural plasticity and inter-regional communication, thereby enhancing cognitive function and mitigating neuroinflammation^70,71^.

Traditionally regarded as a structure primarily responsible for motor coordination, the cerebellum is now increasingly recognized for its integral role in cognitive processing. Emerging evidence suggests that cognitive aging is associated with widespread cerebellar dysrhythmias^72,73^, highlighting the cerebellum’s susceptibility to age-related functional decline^74–76^. Cerebellar circuits are significantly implicated in age-related changes in brain function^77,78^, reflecting the cerebellum’s extensive connectivity across the whole brain through closed-loop networks that support integrative motor and cognitive functions^79^. Resting-state studies have further demonstrated that genetically and epigenetically regulated networks within the neocerebellum contribute to cognitive function in aging populations^80,81^. Consistent with previous hypotheses and empirical data, the present findings suggest that alterations in cerebellar connectivity may serve as a key neural substrate linking normative aging to cognitive decline. Aging is commonly associated with structural deterioration within the cerebellum, including significant volumetric reductions that may impair intra-cerebellar information processing and integration^76,82^. This impairment is reflected in decreased cerebellar connectivity, which may underline observed deficits in cognitive performance. Intriguingly, the present findings suggest that cannabis use may confer a protective effect against such age-associated reductions in cerebellar connectivity. Specifically, cannabis use was associated with increased FC within cerebellar circuits, particularly in regions previously implicated in higher-order motor control and cognitive functioning in older adults. These findings raise the possibility that cannabis may modulate cerebellar network integrity, potentially contributing to the preservation of functional performance in aging populations.

Previous research has proposed that age-related cognitive decline may, in part, stem from alterations in the structure of whole-brain FC^83,84^. Healthy aging may indirectly impact cognitive performance through age-related modifications in FC patterns. In the current study, we observed that, except for the DMN, most within-domain FNC exhibited a declining trend with age. This suggests a reduction in modular organization across the brain and supports the theory that cognitive aging is characterized by decreased network segregation^85,86^. Such segregation refers to the degree to which specific brain regions maintain specialized functional roles, and its reduction is considered a hallmark of age-related functional decline^87^. More importantly, our findings further reveal that cannabis use may exert opposing effects on the FC structure compared to normative aging. Specifically, cannabis use in older adults was associated with increased within-domain FC in subcortical, cerebellar, and sensorimotor networks, along with decreased between-domain FC involving these same regions. This pattern indicates enhanced network segregation, which may support more specialized processing within functional domains while preserving selective inter-network communication—an organization thought to promote efficient cognitive performance^88^. Interestingly, prior research has shown that chronic cannabis use in younger individuals is associated with reduced network segregation, often manifesting as diminished cognitive control, increased mind-wandering, and impaired task-switching^89^. Taken together with our findings, this suggests that cannabis may have age-dependent effects on brain functional organization, potentially due to differences in neurodevelopment, metabolism, immune response, and neurodegenerative processes. We speculate that cannabinoids and endocannabinoids may exert neuroprotective effects during aging by preserving an optimal balance between functional segregation and integration—an essential feature for maintaining specialized processing and efficient information transfer across brain networks. The enhanced cognitive functioning observed in older cannabis users may be partially attributed to cannabisdriven functional differentiation, as reflected in greater network specialization^90^.

The current study contributes to the emerging body of evidence suggesting that cannabis use may confer neurocognitive benefits in older adults by modulating the organization of functional brain networks. The observed effects imply that cannabinoids may exert neuroprotective influences in aging populations, potentially through their regulatory roles in maintaining or enhancing functional brain segregation and integration. These findings align with theories of neural differentiation and compensatory adaptation, which suggest that age-related changes in brain function can be mitigated by the recruitment or preservation of specialized network structures, partly supported by the endocannabinoid system.

### Limitations and Future Directions

The findings of this study should be considered in the context of several potential limitations. First, while the large sample size derived from the UKB represents strength, the observed effect sizes in this study were relatively modest (maximum |Cohen’s d| = 0.0990–0.4231) compared to those reported in smallerscale studies. We acknowledge that such small effect sizes may constrain the immediate clinical applicability of the results. However, it is essential to note that this is consistent with a growing body of evidence in neuroscience indicating that real brain-behavior associations are typically much smaller than earlier literature suggested. Accurately identifying neuroimaging-based biomarkers thus requires large, high-quality datasets with standardized processing pipelines, such as those employed in the present analysis. The field of genomics provides a relevant precedent: its advancement following a reproducibility crisis was aided by large-scale, rigorous data combined with methodological improvements^91^. Looking ahead, future research could benefit from using targeted study designs—such as subgroup stratification—and advanced analytical techniques, like multivariate or multimodal methods, to enhance the interpretability and potential clinical value of small yet meaningful effects.

Second, the present study employed a cross-sectional design, which is inherently susceptible to cohort and period effects^92^. This methodological constraint limits the ability to draw causal or longitudinal inferences regarding changes in cognitive or neurobiological functioning over time. To address this limitation, future investigations could incorporate longitudinal data encompassing multiple time points of both cognitive and neuroimaging assessments. Such designs would enable the validation of cross-sectional findings and support more robust modeling of individual trajectories of cognitive aging in older adults^93^.

Third, the current study focused exclusively on functional neuroimaging markers associated with cannabis use and cognitive aging. However, prior research has also reported structural brain alterations related to cannabis use in aging populations^94–97^. As with functional findings, these structural results are heterogeneous, with studies documenting both increases and decreases in gray matter volume. This variability underscores the need for well-powered, prospective investigations specifically designed to examine the longitudinal effects of cannabis use on brain structure during normative aging. Given the potential for structural and functional imaging to capture overlapping yet distinct aspects of cannabis-related neural alterations, an integrated multimodal approach is warranted. Future studies should leverage advanced data fusion methodologies in conjunction with mediation analysis to elucidate the mechanistic pathways linking cannabis use to cognitive outcomes via multimodal brain changes.

Fourth, in contrast to findings from studies involving younger populations, the present results suggest an opposite pattern of association between cannabis use and cognitive function in older adults, as evidenced by positive correlations between cannabis use and cognition in terms of whole-brain FC. However, a limitation of this study is the restricted age range of the UKB sample (participants aged > 45 years), which precludes a direct investigation of age-dependent effects or developmental trajectories. Understanding whether and how cannabis use exerts differential effects across the lifespan requires data encompassing a broader age spectrum. Large-scale longitudinal neuroimaging initiatives—such as the Adolescent Brain Cognitive Development (ABCD) Study and the Human Connectome Project (HCP)—provide critical resources for investigating both neurodevelopmental and neurodegenerative trajectories in relation to cannabis exposure. Our research group has previously applied functional connectivitybased analytic approaches to these developmental datasets, offering a strong methodological foundation for future integrative analyses^35,36,40,98^. By integrating data from ABCD, HCP, and UKB, future research can develop a comprehensive lifespan model to characterize the age-specific neural and phenotypic consequences of cannabis use and explore potential causal mechanisms underlying these effects.

## Supplementary Files

This is a list of supplementary files associated with this preprint. Click to download.

- Supplementarymaterial.docx

## Figures and Tables

**Table I.** Demographics of subjects passed QC

Basic Demographics	Cannabis users	Non-users
**Total subject**	5488	18482
**Age** (year)	60.93 ± 7.02	64.24 ± 7.44 *
**Sex** (F/M)	2703/2785	10699/7813 *
**Cannabis Usage** (light users/heavy users)	3839/1649	NAN
**Mean FD** (mm)	0.1760 ± 0.0591	0.1822 ± 0.0601 *
**Tower rearranging** (sub)	0.77 ± 0.16 (3659)	0.73 ± 0.18 (11586) *
**Matrix pattern completion** (sub)	0.63 ± 0.18 (3670)	0.59 ± 0.17 (11167) *
**Paired associate learning** (sub)	7.62 ± 2.38 (3693)	6.99 ± 2.59 (11779) *
**Fluid intelligence** (sub)	7.01 ± 1.37 (3788)	6.70 ± 1.46 (12123) *
**Picture vocabulary** (sub)	7.22 ± 2.00 (5204)	6.71 ± 2.03 (17146) *
**Numeric memory** (sub)	0.42 ± 0.08 (3676)	0.40 ± 0.08 (11656) *

F = Female; M = Male; FD = Framewise Displacement; mm = Millimeter; sub = Subject Number;

* indicates significant difference between groups (p < 0.0001).

## Data Availability

Data used in the preparation of this article were obtained from the UKB database (https://ams.ukbiobank.ac.uk/) via the application ID 34175: Identify biomarkers for distinguishing different mental disorders using brain images and their associations with genetic risk.
